# High Boron Content Enhances Bioactive Glass Biodegradation

**DOI:** 10.3390/jfb14070364

**Published:** 2023-07-11

**Authors:** Amina Gharbi, Hassane Oudadesse, Hafedh el Feki, Wissem Cheikhrouhou-Koubaa, Xanthippi Chatzistavrou, Julietta V. Rau, Jyrki Heinämäki, Iulian Antoniac, Nureddin Ashammakhi, Nabil Derbel

**Affiliations:** 1 CEM Lab, National Engineering School of Sfax, Sfax University, Sfax 3018, Tunisia; 2 LT2S Lab, Digital Research Centre of Sfax, Technopole of Sfax, P.O. Box 275, Sfax 3000, Tunisia; 3 ISCR, University of Rennes 1, UMR CNRS 6226, 35042 Rennes, France; 4 Faculty of Sciences of Sfax, Sfax University, Sfax 3018, Tunisia; 5 Department of Chemical Engineering and Material Science, College of Engineering, Michigan State University, East Lansing, MI 48824, USA; 6 Istituto di Struttura della Materia, Consiglio Nazionale delle Ricerche (ISM-CNR), Via del Fosso del Cavaliere 100, 00133 Rome, Italy; 7 Department of Analytical, Physical and Colloid Chemistry, Institute of Pharmacy, I.M. Sechenov First Moscow State Medical University, Trubetskaya 8, 119991 Moscow, Russia; 8 Institute of Pharmacy, Faculty of Medicine, University of Tartu, Nooruse 1, 50411 Tartu, Estonia; 9 Faculty of Material Science and Engineering, University Politehnica of Bucharest, SIM 313, 060042 Bucharest, Romania; 10 Institute for Quantitative Health Science and Engineering, Department of Biomedical Engineering, College of Engineering and College of Human Medicine, Michigan State University, East Lansing, MI 48824, USA; 11 Department of Bioengineering, University of California, Los Angeles, CA 90095, USA

**Keywords:** borosilicate bioactive glass, boron, degradation, physico-chemical characterizations, hydroxyapatite layer

## Abstract

Derived Hench bioactive glass (BaG) containing boron (B) is explored in this work as it plays an important role in bone development and regeneration. B was also found to enhance BaG dissociation. However, it is only possible to incorporate a limited amount of B. To increase the amount of B in BaG, bioactive borosilicate glasses (BaG-B_x_) were fabricated based on the use of the solution-gelation process (sol-gel). In this work, a high B content (20 wt.%) in BaG, respecting the conditions of bioactivity and biodegradability required by Hench, was achieved for the first time. The capability of BaG-B_x_ to form an apatite phase was assessed in vitro by immersion in simulated body fluid (SBF). Then, the chemical structure and the morphological changes in the fabricated BaG-B_x_ (x = 0, 5, 10 and 20) were studied. The formation of hydroxyapatite (HAp) layer was observed with X-ray diffraction (XRD) and infrared (IR) spectroscopy. The presence of HAp layer was confirmed using scanning electron microscopy (SEM) and transmission electron microscopy (TEM). Enhanced bioactivity and chemical stability of BaG-B_x_ were evaluated with an ion exchange study based on Inductively Coupled Plasma–Optical Emission Spectrometry (ICP-OES) and energy dispersive spectroscopy (EDS). Results indicate that by increasing the concentration of B in BaG-Bx, the crystallization rate and the quality of the newly formed HAp layer on BaG-B_x_ surfaces can be improved. The presence of B also leads to enhanced degradation of BaGs in SBF. Accordingly, BAG-B_x_ can be used for bone regeneration, especially in children, because of its faster degradation as compared to B-free glass.

## 1. Introduction

Bone defects may result from congenital deficiencies, trauma, infection, tumors, or surgical removal [1,2]. In children, healing is faster than in older patients, and it follows a different pattern, e.g., in the skull, bone is laid out externally and resorbed from the inner side of the skull [3]. Therefore, the development of biodegradable devices that suit the pace and mode of bone healing in children was explored [4,5,6].

Biodegradable polymers could be potential candidates; however, they lack osteoconductivity and often lead to chronic inflammatory reactions [7]. Therefore, bioceramics and polymer ceramic composites have been explored [8]. The development of bioceramics and bioactive glass (BaG) in particular [9] has proven to be effective in promoting bone healing and regeneration both in experimental and clinical settings [10]. BaG induces a biological response which results in the formation of a chemical bond between the tissue and the biomaterial [11,12]. This bond is formed by a biologically active hydroxyapatite (HAp) layer that forms on the surface of the biomaterial following its implantation, promoting bonding with host tissues [13]. There are different types of BaG, the first that was developed by Hench, the 45S5 Bioglass^®^ (45 wt.% SiO_2_, 24,5CaO, 6P_2_O_5_ and 24,5Na_2_O) [14]. Larry Hench started by studying the SiO_2_-CaO-Na_2_O ternary diagram in which he systematically added 6% by mass of P_2_O_5_ [15]. However, bioactivity and biodegradability were only observed for certain compositions, which limit the variations in the proportions of SiO_2_, CaO, and Na_2_O [9]. In vitro tests have shown that 45S5 Bioglass^®^ causes a carbonated HAp surface layer formation. This layer is chemically and structurally similar to bone-carbonated HAp and allows a direct bond between tissue and implant.

To enhance the degradation of Bioglass^®^ and make it suitable for applications in pediatric patients, borate-based BaGs (BaG-Bs) were developed [16,17,18,19]. This was achieved through the replacement of SiO_2_ with B_2_O_3_ in the Bioglass^®^ (45S5), respecting the conditions of bioactivity and biodegradability required by Hench [14], and derived glasses demonstrated acceptable bioactivity in vitro [20,21] with the formation of an HAp layer [22]. In particular, pure BaG-B has recently been explored by Richard, who revealed that not only can bone regeneration be enhanced using BaG-B, but also a faster rate of BaG degradation could be achieved, as compared to silicate (SiO_4_)-based 45S5 Bioglass^®^ [23]. It was found that (BO_3_) analogous to 45S5 Bioglass^®^, in which all SiO_2_ was replaced with B_2_O_3_ (B-45S5), is totally converted to HAp during the period of 3–4 days, in vitro. This is much faster than (SiO_4_) 45S5 glass (Si-45S5), in which the process takes several weeks to convert only 50% of it into an HAp layer [24,25,26,27]. However, for each clinical application a suitable lifetime of the implant is required. In addition to its low mechanical strength and density, borosilicate glass is also non-biodegradable due to its high B content (36%) [28,29,30].

Several researchers studied possibilities to control the degradation rate, mechanical properties, and electrical and thermal behaviors of BaGs by adjusting their initial chemical composition and with the introduction of various other oxides such as B_2_O_3_, CaF_2,_ and Al_2_O_3_ in BaG [31,32,33]. Different chemical compositions of BaG-B have been applied as scaffolds in maxillofacial applications or as coatings of orthopedic implants [34,35]. For instance, the degradation rate and the bioactivity behavior in vitro of BaG-B were evaluated [36]. Recently, Saranti et al. mentioned that B incorporation in the glass matrix of CaO–B_2_O_3_–P_2_O_5_ system has a catalytic effect on enhancing bioactivity [37]. In this context, Lee et al. proved that adding B enables the fabrication of implants suitable for maxillofacial applications due to their low durability in physiological fluids [38,39]. Additional interesting biological effects of B also include the promotion of extracellular matrix (ECM) regeneration [40], stimulation of wound healing in vivo, and contribution to bone remodeling [41]. More recently, another study reported that cancellous screws coated with BaG-B could bond easily to cancellous bone and advance bone-implant osseointegration [42], without showing cytotoxicity [43]. However, studies cited above reported weak mechanical properties of developed products, and they were limited to bone-coating applications. For example, for B_2_O_3_-NaCaPO_4_–SiO_2_–PO_4_ BaG composition, several physical and mechanical tests proved that the introduction of B in the BaG matrix decreases density and microstructure. Additionally, B reduces the modulus of rigidity and hardness of BaG [44].

Therefore, the aim of the current study is to develop and investigate in vitro a resorbable sol-gel-derived BaG-B having different amounts of B added into a free-boron glass (BaG-B_0_) network to provide the bioactivity and biodegradability required to meet a wide range of orthopedic and maxillofacial application needs. After soaking in simulated physiological liquid (SBF), physico-chemical bonds are formed at the interface of BaG-B_x_ and SBF resulting in the development of an HAp layer. Depending on the amount of incorporated B, morphological changes have been revealed for this newly formed crystallized layer.

This study faces the challenge of substituting Si for B in a BaG matrix with a maximum amount that has never been studied before, while maintaining constant concentrations of other system modifiers (sodium (Na), phosphate (P), and calcium (Ca)), as well as maintaining the vitreous structure so that bioactivity, resorbability, and mechanical properties are optimized according to previously studied compositions [45].

**Figure 1:**
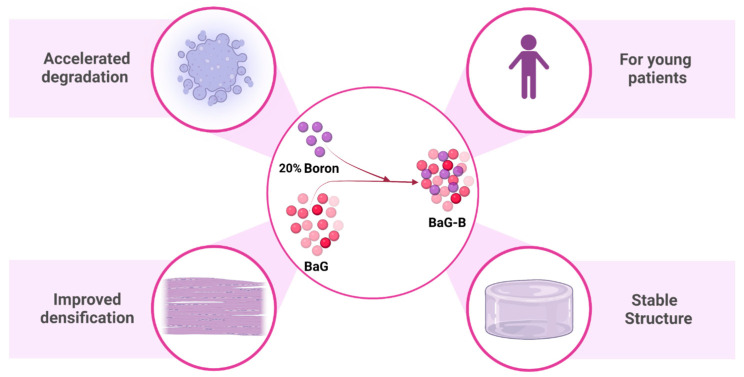
Increasing boron content (B) in bioactive glass (BaG) to 20% results in improved morphological and structural properties and accelerates the degradation of borosilicate bioactive glass (BaG-B). This figure was produced using Biorender.com.

Obtained BaG-B was analyzed for its structure, morphology, and ion exchange after in vitro simulation in SBF to better understand the resorbability rate and bioactivity process of a new class of BaG-B that can promote efficient osteoconduction in bone defects in children, because their skeleton is growing and it is better to avoid the use of permanent implants that may interfere with skeletal growth and imaging (Figure 1).

## 2. Materials and Methods

### 2.1. Glass Preparation

BaG material (SiO_2_–CaO–Na_2_O–P_2_O_5_ system) was prepared using the sol-gel method, according to a previously described protocol [46]. Borosilicate BaG (BaG-B) with varying B content was then produced (Table 1).

**Figure 2:**
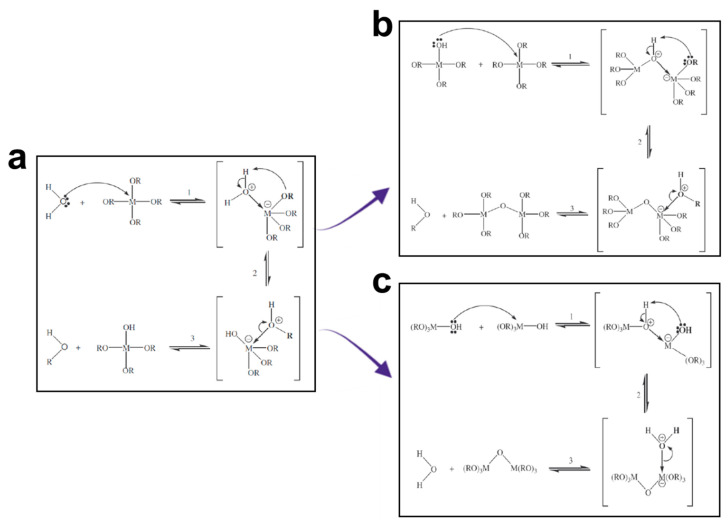
Molecular schematic of the synthesis used to produce borosilicate bioactive glass (BaG-B); with M = Si, B and R = Na, Ca, P. (**a**) Hydrolysis mechanism of BaG-B which results in two parallel internal processes: alkoxolation and oxolation. (**b**) Polycondensation mechanism (alkoxolation). (**c**) Polycondensation mechanism (oxolation).

To fabricate BaG-B_x_, stoichiometric amounts of tetraethyl orthosilicate (Si(OC_2_H_5_)_4_: TEOS) (Fluka, Buchs, Switzerland), triethylphosphate (TEP) (OP(OC_2_H_5_)_3_ (Eastman, Tennessee, USA), calcium carbonate CaCO_3_ (Merck, Darmstadt, Germany), sodium carbonate Na_2_CO_3_ (Sigma Aldrich, St. Louis, MO, USA), and boron oxide B_2_O_3_ (Sigma Aldrich, Missouri, USA) were added to a 2N aqueous acetic acidic solution, under magnetic stirring. After the addition of each of the above-mentioned reactants, the solution was stirred for one hour. The resultant sol was introduced into a cylindrical Teflon beaker for hydrolysis and then condensation at room temperature for six days to achieve a homogenous powder at the molecular level [48]. Chemical mechanisms involved in both steps of sol-gel synthesis are presented in Figure 2:

Formed gel (Figure 2b,c) was then kept at 70 °C for three days and dried at 150 °C for 52 h. The resulting dried gel was then analyzed with thermal analysis, under nitrogen, to identify the lower temperature required to decompose the initial salts into the final oxides. Thermo-gravimetric and differential scanning calorimetry (TG/DSC, Labsys 1600 TGDTA/DSC, Setaram, Lyon, France) analyses showed that a thermal stabilization of the dried gel is achieved at a temperature below 520 °C. Obtained dried gel was then thermally treated at 500 °C for 3 h under vacuum with a heating rate of 0.5 °C min^−1^.

### 2.2. In Vitro Assays

**Figure 3:**
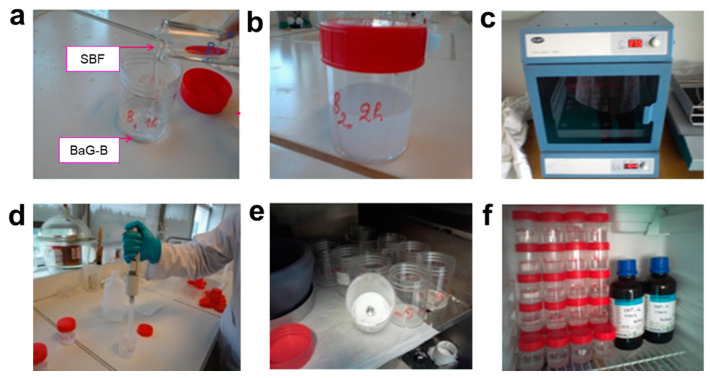
Immersion manipulation of borosilicate bioactive glass (BaG-B) powders in simulated body fluid (SBF). (**a**) BaG-B_x_ were immersed in SBF placed into sealed polyethylene bottles. (**b**) Both solid and liquid phases were properly mixed. (**c**) Mixtures were placed in an incubator. (**d**) SBF solution extraction to wash out powder. (**e**) Dried powders for structural characterization. (**f**) SBF solutions for chemical characterization.

BaGs were immersed in SBF, which was synthesized in this study according to Kokubo’s method [49]. After various immersion periods, BaG-B_x_ were retrieved from SBF, washed with de-ionized water, and dried at room temperature. Then BaG-Bx samples were maintained at 37 °C under controlled agitation (50 rpm) for short (1, 2, 4, 8, 10, 12, and 16 h) and long (1, 2, 5, 10, 15, and 30 days) follow-up times (Figure 3). In vitro assays were performed under static conditions with a ratio of BaG to SBF volume equal to 0.5 mg/mL (30 mg/60 mL). SBF was not changed during the experiment. In vitro bioactivity and chemical stability of BaGs were assessed by study of ion exchange. Structural and chemical characterizations were performed for each BaG sample after each time point as detailed in the following sections.

### 2.3. Structural Characterization

#### 2.3.1. X-ray Diffraction (XRD)

Determination of the nature of the neo-formed layer of BaG-B_x_ was studied using AXS D8 ADVANCE diffractometer (BRUKER, Billerica, MA, USA), with voltage U = 30 kV and current I = 20 mA. The X-ray cannon used has a copper cathode producing radiation (Kα_1_ +K α_2_). The radiation used is monochromatic with wavelength l = 1.5406 Å.

#### 2.3.2. Infrared Spectroscopy (IR)

Infrared spectroscope Equinox 55 (BRUKER, Billerica, MA, USA) at the range of 2000–400 cm^−1^ was applied to determine the structure of the new layer on the BaG surface.

#### 2.3.3. Scanning Electron Microscopy-Energy-Dispersive Spectrometry (SEM-EDS)

Scanning electron microscopy (SEM, Joel JFC 1100, ZEISS, Stuttgart, Germany) was used for the evaluation of BaG-B microstructure, after the immersion of BaG-B_x_ samples for different periods of time ranging from 1 h to 30 days. Energy-dispersive spectrometry (EDS) was used for the chemical analysis of BaG surfaces. Samples for SEM and EDS were covered with a gold–palladium layer to allow surface conduction. Three fields in each sample were examined using SEM and EDS (one samples for each BaG-B composition).

#### 2.3.4. Transmission Electron Microscope (TEM)

A high-resolution imaging at the nanoscale was performed using a transmission electron microscope (TEM, JEOL JEM-2010, Ltd., USA), which was equipped with a lanthanum hexaboride (LaB_6_) tip gun and a GATAN Dual Vision camera (1300 × 1000 pixels), and the electrons were accelerated under a maximum voltage of 200 kV.

### 2.4. Chemical Characterization

Elemental concentrations of SBF (Si, Ca, P, B) were investigated using Inductively Coupled Plasma–Optical Emission Spectrometry Instrument (ICP-OES, Agilent 5800, LabX, Santa Clara, CA, USA) for short- (1, 2, 4, 8, 10, 12, and 16 h) and long-term (1, 2, 5, 10, 15, and 30 days) follow-up times after immersion in SBF.

## 3. Results and Discussion

### 3.1. Physico-Chemical Characterizations after Immersion in SBF

#### 3.1.1. XRD

**Figure 4:**
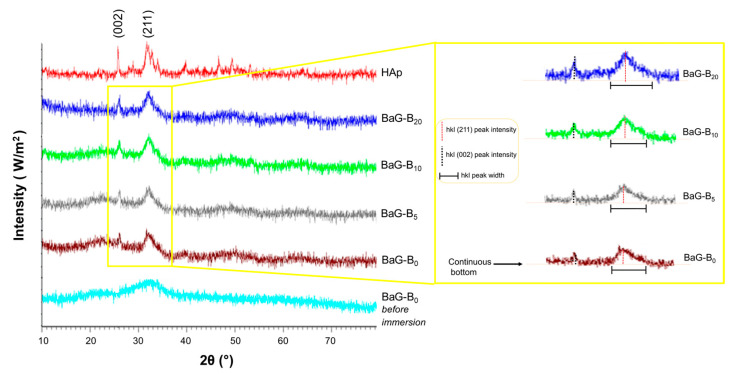
X-ray diffractograms of borosilicate bioactive glasses (BaG-B_x_) after 30 days of immersion in simulated body fluid (SBF).

X-ray diffraction patterns of the surfaces of BaG-B_x_ (0 ≤ x ≤ 20, in which x is the weight percentage of B_2_O_3_ intercalated in glass matrix) after 30 days of immersion in SBF showed the typical characteristic of crystalline apatite phase (Figure 4). For reference, a diffractogram of synthetic HAp was used [50,51].

Profiles showed that two crystal structures of BaG-B_x_ and reference glass BaG-B_0_ are iso-typical. Indeed, broad diffraction lines of HAp formed on the BaG particle surfaces at around 26° and 32° in 2θ, corresponding to the (002) and (211) planes, respectively, confirmed the bioactivity of BaG-B chemical compositions. However, with increasing B content, diffraction lines were more intense, narrower, and brighter, which is characteristic of crystallized powders. No other crystalline phase was detected with XRD.

Phases, which are usually observed for silicate hydroxyapatite (Si-HAp), tricalcium phosphate (TCP), silicocarnotite [52], or limes (CaO, CaCO_3_) [53] were not detected in the current study. It is also noteworthy that there was a 2θ shift for diffraction peak located at 26° of the BaG-B_x_ compared to that of BaG-B_0_. Thus, the angular gap between the peaks located in the 25–27° region was narrow.

These angular variations were synonymous with a slight modification of distance between two diffraction planes (002), probably due to the presence of silicates (SiO_4_) and borates (BO_3_) in the newly formed HAp mesh. The HAp layer is the main mineral constituent of bone. Moreover, its formation is based on an ion exchange mechanism between the glass surface and the body fluids.

X-ray diffraction pattern superposition of BaG-B_0_ glass powder, and BaG-B_x_ revealed a significant difference. This could be due to a progressive increase in B in the glass matrix, which was indicated by the sharpness of the peaks and the increase attributable to the reflections (0 0 2) and (2 1 1) (Figure 4). These effects can be explained not only by considering the stability and non-distortion of the SiO_4_ network generated in the structure of BaG-B_x_ due to the substitution of SiO_4_ groups with BO_4_ groups, but also by the presence of carbonate (C-O) in this HAp layer [54]. After immersion in SBF, morphological modification by the addition of B to the glass leads to a preferential growth mechanism toward crystallization direction (002) and (211). This depends on the duration of immersion in SBF and the chemical composition of the fabricated BaG-B_x_. Thus, the formation of crystal needles in BaG structure seems to be favored by the substitution of silicate ions (Si^4+^) with borate ions (B^4+^) [38].

A similar crystallization evolution on XRD was observed by other investigators when B was added to Hench Bioglass ^®^ 45S5 [55]. XRD allows us to better understand the evolution of the BaG-B/SBF system and to detect any structural changes generated on the surface of BaGs after their immersion in the physiological medium.

#### 3.1.2. Infra-Red Spectroscopic Analysis

**Figure 5:**
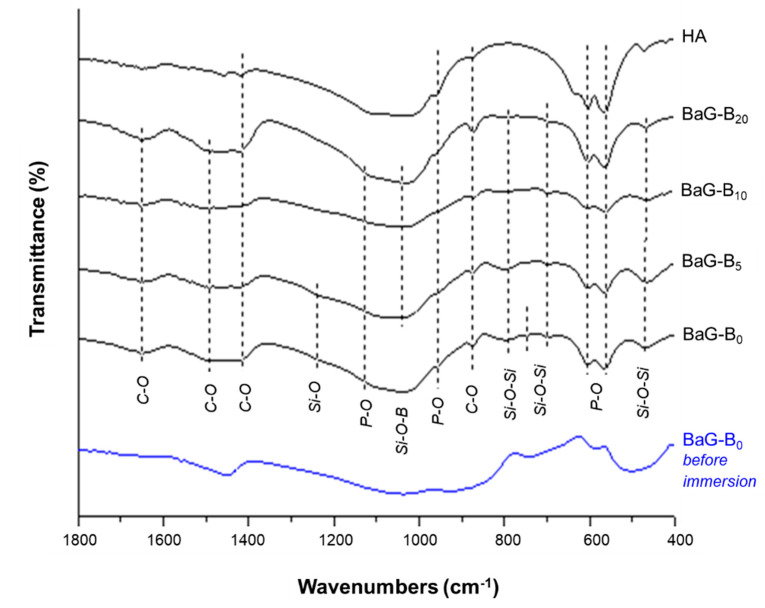
Infrared spectra of borosilicate glasses after 30 days of immersion in simulated body fluid (SBF).

After immersion, IR allows us to study the effect of B incorporation within the glass network on the silicate, phosphate, and carbonate bands. Results confirm the formation of an apatite layer on the surface of BaG-B glass after 30 days of immersion (Figure 5).

Characteristic IR bands of vibrational modes of ionic groups in P-O bands (Table 2) [56], silicate phosphocalcic HAp (Table 3) [57,58,59], and C-O bands (Table 4) [60,61] compiling with previously reported results were seen.

In general, these glasses showed the majority of the absorption bands characteristic of a carbonated HAp. By definition, “labile” carbonates are clusters that would occupy poorly organized sites in the HAp crystal and would therefore be more reactive than carbonates occupying better-organized sites [62] or atmospheric CO_2_ adsorbed on the surface of powders.

The presence of bands for the frequency of the antisymmetric deformation (υ_4_ = 562 cm^−1^ and 603 cm^−1^) and symmetric elongation (υ_1_ = 960 cm^−1^) modes of PO_4_^3-^ ions was confirmed for BaG-B glasses after 30 days of immersion in SBF (Table 2). In addition, the last vibration band of PO_4_ (υ_1_ = 960 cm^−1^) became narrower and more intense for BaG-B_20_. As a function of B content, clear modifications of vibrational bands attributable to the phosphate ions occurred. In particular, the increase in the intensity of the band located at 1121 cm^−1^ is related to environment change (isomorphic ionic exchange between PO_4_ groups and SiO_4_ and/or BO_4_ groups, Figure 5). Consequently, P ion affinity to BO_4_ group is higher than to SiO_4_ group, which facilitates rapid formation of a phosphocalcic HAp layer on the BaG-B surface. The key characteristic of BaG is its ionic atmosphere’s occurrence that does not exist in crystallized material when immersed in the physiological environment. These ionic surroundings have been attributed to the surface appearance of an HAp crystal layer. Moreover, Si-O-Si vibrational band observation of around 475 cm^−1^ is clear regardless of the amount of B incorporated in the BaG matrix. The band at approximately 458 cm^−1^ is assigned to Si-O-Si and O-Si-O bending modes of bridging oxygen (Q^4^) atoms overlapped with B-O-B linkages [63]. For compositions richer in B (BaG-B_10_; BaG-B_20_), a band disappearance of υ_1_ vibration mode frequency attributed to SiO_4_ cluster (Table 3) was proven. Indeed, a similar decrease until intensity disappearance was observed due to B incorporation attributed to other silicate-specific vibrations located at 699 cm^−1^, 794 cm^−1^, and at 1235 cm^−1^ designated to Si-O in silica [57,64], and Si-O vibration mode (Table 3), respectively. In addition, the vibrational band located at 1045 cm^−1^, probably attributed to Si-O-B cluster, becomes relatively less broad and more intense in the samples that contain 20% B_2_O_3_.

The influence of B incorporation on BaG vibrational spectra was confirmed by demonstrated proportional correlation between XRD results (Figure 4).

The changes reported for SiO_4_- and PO_4_ -specific vibrations in BaG structure after immersion in SBF are due to the vibrational component modifications of SiO_4_/BO_4_ and/or BO_3_ groups in their environment (modification of local interactions). These modifications could be related to strong interactions between these groups leading to the “extinction” of certain vibrations or incorporated B content. Infrared spectrometry allows us to affirm that B is integrated in silicate apatite structure, when B_2_O_3_ content is 5 wt.% or more, as demonstrated by the appearance of a weak crystalline phase.

However, after 30 days of immersion in SBF, IR spectra indicate that all samples (with or without B) show the presence of carbonate groups in the majority (vitreous) and minority (crystalline apatite) structures (Figure 5). The carbonation of boron-free BaG (BaG-B_0_), thus BaG-B_x_, can be explained by the high atmospheric concentration of CO_2_, which is partly adsorbed on the surface of the glass particles and also dissolved in the SBF. At the same time, CO_3_^2−^ ions are among the main components of carbonate (HCO^−^_3_) physiological solution. Despite precautions taken in this study, carbonation remains inevitable.

IR spectra of produced compounds showed bands around 1492 and 1651 cm^−1^ attributed to the C-O group. Moreover, the formation of a carbonated HAp layer is proved by the clear appearance of bands at 873 and 1410 cm^−1^ specific to CO_3_^2-^ groups in the apatite structure (Table 4). IR patterns show the presence of CO_3_^2-^ bands in the apatite phase formed on the BaG particle surface. This is evidenced by the angular shift 2θ [65,66] (Figure 4). These results also explain the effect of incorporating CO_3_ and BO_4_ and/or BO_3_ groups in the BaG structure, and highlight the incorporation mechanisms of BO_3_/BO_4_ and CO_3_ groups in the amorphous and apatite structure during immersion. For this purpose, it can be assumed that the layer formed on the BaGs surface after immersion in SBF is a layer of phosphocalcic HAp co-substituted in carbonate (CO_3_) and silicate/borate (SiO_4_/BO_4_) [67]. Accordingly, the incorporation of B in the BaG network leads to enhanced bioactivity of BaG, as it was evidenced by the sharpness and increase in the band intensity of vibrational modes designated to phosphate (PO_4_) and carbonate (CO_3_) groups characteristic of a crystallized apatite layer [68].

#### 3.1.3. BaG-Bx Surface Modifications

**Figure 6:**
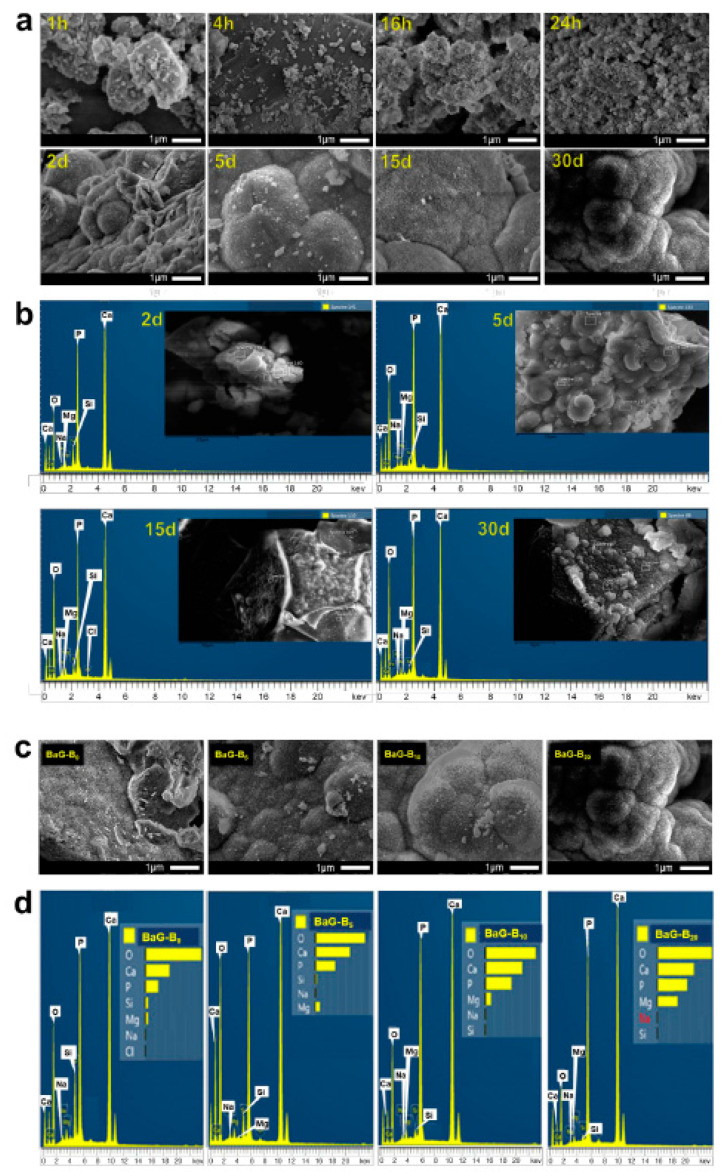
Microstructural observation and chemical analysis with scanning electron micrography- energy dispersive spectrometry (SEM-EDS) for borosilicate bioactive glasses (BaG-B_x_). (**a**) SEM micrograph of BaG-B_20_ powder surface as a function of SBF immersion time. (**b**) Zone analysis of BaG-B_20_ powder surface after 2, 5, 15, and 30 days of immersion. (**c**) Micrographs of BaG-B_x_ powders (×10.000) after 30 days of immersion in SBF. (**d**) EDS spectra of BaG-B_x_ powders after 30 days of immersion.

After one month of immersion, the presence of lamellae and filaments of angular shape and heterogeneous size on the BaG-B_x_ particle surface [69] was proved for BaG-B_20_ (Figure 6a). This observation is in agreement with the XRD results of the current study, which showed that the BaG-B_20_ surface had an HAp layer at all immersion time points, being more at longer follow-up times. By 30 days of immersion, the HAp layer became thicker and had more organized crystals (Figure 6c). With increasing B content from 5 to 20% in BaG, the HAp layer became more consolidated. For example, the surface of BaG-B_20_ samples was completely covered with a crystallized Hap layer, which can be explained by the fact that a high content of B is easily released to SBF solution causing the creation of new active groups capable of accelerating ion exchange between BaG and SBF.

Going from 1 to 4 h of immersion in SBF, the surface of BaG-B_20_ particles exhibited a layer of Hap (Figure 6a) similar to that seen with B-free glass (BaG-B_0_) immersed in SBF for 30 days (Figure 6c). The crystals formed in Hap of BaG-B_20_ were smaller, more regular, and finer than those formed on the surface of BaG-B_0_ particles (Figure 6c), demonstrating a better crystallinity of BaG-B_20_. As it is well known, chemical reactivity of minerals is related to their surface properties. Our previous recently published study proves that B presence enhances BaG-B_x_ densification [45]. A good understanding of the BaG-B_x_ morphology leads us to assess the in vitro properties. For BaG-B_20_, after 16 h of immersion in SBF, it exhibited an irregular surface (Figure 6a), which was only observed after 30 days of immersion for BaG-B_0_ (Figure 6c). After two days of immersion in SBF, the BaG-B_20_ surface was covered with crystals. On the other hand, the crystallized layer on BaG-B_0_ was observed only after 30 days of immersion in SBF. Thus, the solubility of BaG was improved with increasing B content in BaG (Figure 6c). Additionally, the surface free energy of silicate (SiO_4_), borate (BO_3_), carbonate (CO_3_), and phosphate (PO_4_) clusters plays an important role in the solubility of BaG-B_x_ in physiological solution, since BaG solubility rates are dependent on these intermolecular energies.

The distortion of the vitreous network leads to ion formation in the surface of BaG particles, and increases ion activity energy. These ions (unsaturated atoms) in the mineral medium of BaG/SBF have a strong capability to attach and/or repulse other chemical entities. This represents the driving force behind chemisorption and ionic exchange between SBF and BaG, which leads to its dissociation, allowing surface functional groups to appear [70]. Furthermore, the chemical composition of BaG determines its ionic activity, and both of these affect its solubility [38]. In this sense, the formation of an HAp surface layer controls the solubility of glass. The HAp layer contains mineral labile ions (CO_2_^3−^, HPO_4_^2−^ and PO_3_^4−^).

This phenomenon suggests that some reactions take place at the BaG-B_x_/SBF interface during immersion. Because of its planar geometry, BO_3_ facilitates these reactions, which may be limited to preferential adsorption of calcium and/or phosphate ions. However, solubility can result in a diffusion barrier that ultimately prevents full crystallization of the BaG-B_x_ surface.

In the current study, EDS showed that silicon (Si), calcium (Ca), phosphorus (P), and sodium (Na) were detectable at each time point. Regardless of immersion time, signals of Ca and P peaks were the most intense (Figure 6b). It was found that Si concentration decreases with time, which confirms its release to SBF. After one hour of immersion, B was not detectable in BaG, which explains its high solubility and its fast release to the SBF physiological solution (Figure 6b). EDS showed that after 30 days, BaG-B_20_ was biphasic (silicate amorphous and crystallized phases) due to the high content of Ca (as indicated by high EDS peak signal intensity, which is slightly stronger than that of P, Figure 6b). This means that for the same chemical composition of BaG-B_x_, the transfer kinetics of some ions during immersion depends only on contact time between BaG and SBF.

In addition, solid-to-liquid ratio efficiency (BaG mass/SBF volume) is a very important factor. It allows the control of chemical reactivity and crystallization kinetics and quality. It is possible to precisely explain results observed in the current study based on the published literature on ion transfer mechanisms [71,72]. Different factors were hypothesized to cause BaG solubility, including high chemical affinity of the BaG surface to certain ions (P, Ca), the nature of clusters present in physiological solution, bonded interactions between particles at the onset of immersion until saturation, and choice of BaG chemical composition [71,72]. Accordingly, adding B accelerates the growth of crystalline particles on the surface of BaG particles [40]. EDS analysis of the BaG-B_x_ surface after 30 days of immersion in SBF confirms the growth of an amorphous calcium phosphate layer on the surface of BaG (Figure 6d). Compared to the EDS spectrum obtained for BaG-B_0_, a significant decrease in Si quantity according to added B amount was observed (Figure 6d). On the contrary, quantities of P and Ca have evolved according to the increase in added B amount. This confirms once again the better calcium phosphate layer crystallization for BaG-B_20_. In addition, EDS patterns showed clearly increasing magnesium (Mg) concentration in BaG with increasing B content (Figure 6d). It is well known that Mg is an essential element for human metabolism, and it is naturally present in bone matrix [73,74]. In addition, Mg helps to stimulate bone tissue growth [75,76,77].

#### 3.1.4. Nano-Structural Changes in BaG-B

**Figure 7:**
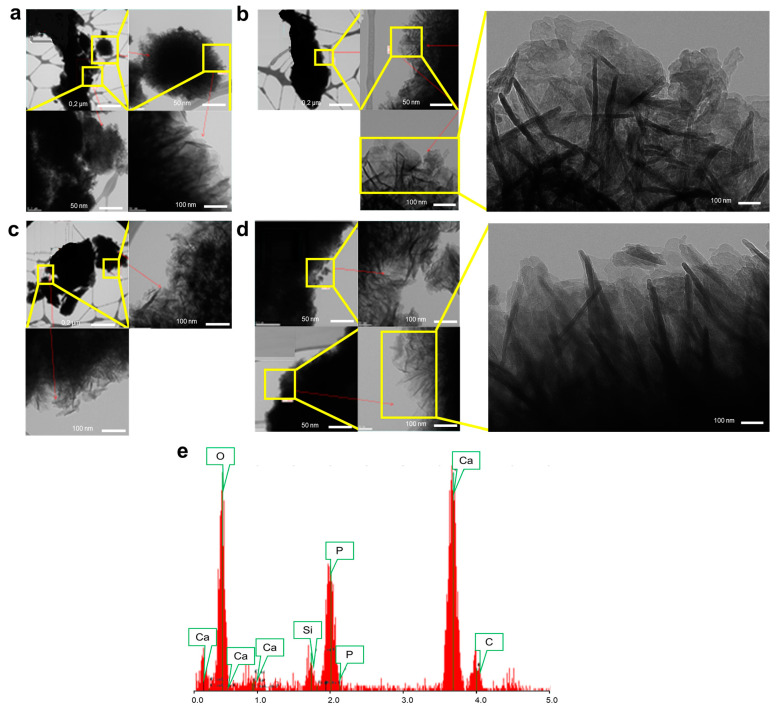
Transmission electron micrograph (TEM) images, at varying magnifications of borosilicate bioactive glass (BaG-B_x_) after 30 days of immersion in simulated body fluid (SBF). (**a**) TEM micrograph of BaG-B_0_. (**b**) TEM micrograph of BaG-B_5_. (**c**) TEM micrograph of BaG-B_10_. (**d**) TEM micrograph of BaG-B_20_. (**e**) Energy dispersive spectrometry (EDS) spectra of BaG-B_20_ after 30 days’ immersion in SBF.

An HAp layer formed on the surfaces of BaG-B_x_ samples after 30 days of immersion in SBF (Figure 7). The incorporation of B in the BaG structure leads to a dilation in its vitreous network (formation of BO_3_ group instead of SiO_4_), which results in decreased durability of BaG within SBF. Crystal growth in BaG-B_x_ after one month of immersion in SBF occurred in two different geometrical forms, in platelets and in fibers (needles), according to preferred directions [78] (Figure 7a–d). Therefore, the dissolution rate of BaG-B_20_ (Figure 7d) was faster than that of BaG-B_5_ (Figure 7b). This proves that B ions could act as activators of HAp nucleation and crystallization.

EDS analysis of the crystallized surface of BaG-B_20_ demonstrated that high B content influences BaG tendency to dissolve and favors exchange of Ca, P, and O ions between BaG and SBF (Figure 7e). As a result, high content of these ions in the BaG was observed on EDS (Figure 7e), which was also confirmed by the spectroscopic results (Figure 5).

#### 3.1.5. Ion Exchange between BaG-B and SBF

**Figure 8:**
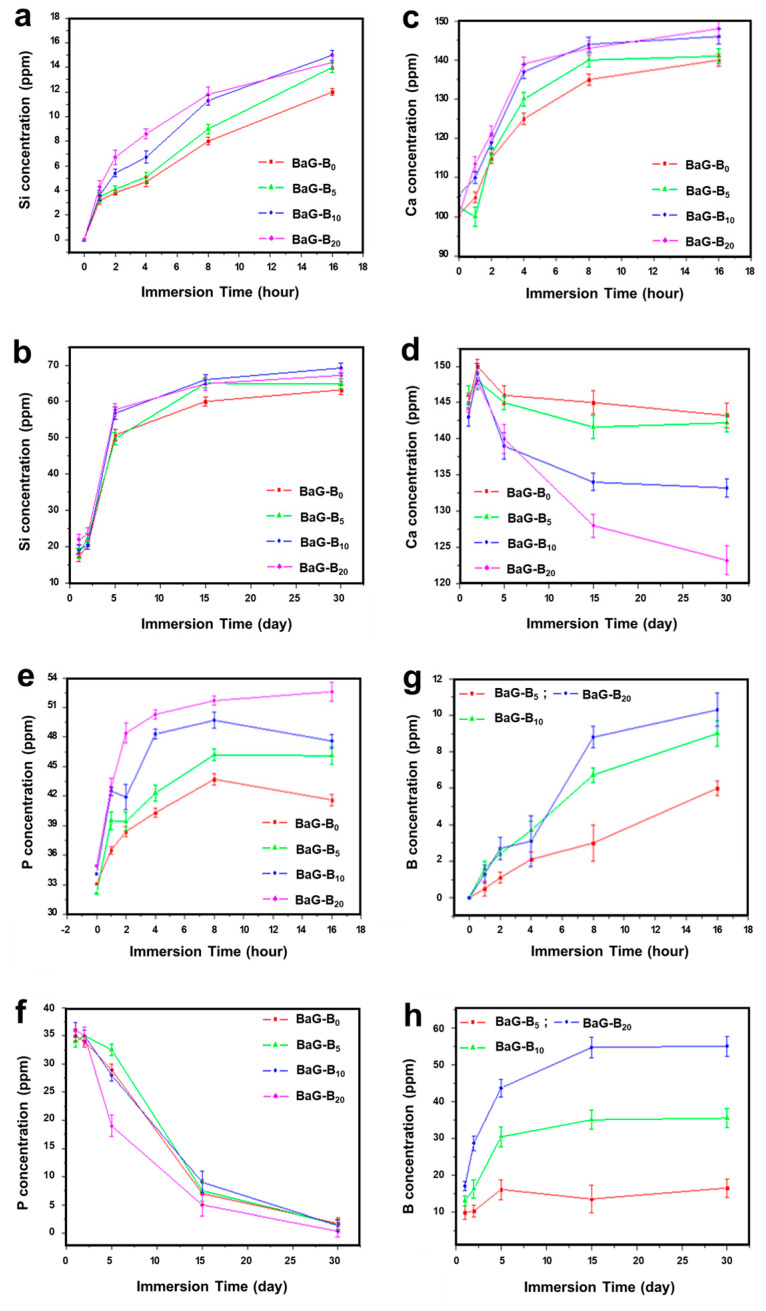
Composition of simulated body fluid (SBF) after immersion of borosilicate bioactive glasses (BaG-B_x_) at short times (curves on top) and at long times (curves below). (**a**) Silicon (Si) concentration at short time. (**b**) Si concentration at long time. (**c**) Calcium (Ca) concentration at short time. (**d**) Ca concentration at long time. (**e**) Phosphorus (P) concentration at short time. (**f**) P concentration at long time. (**g**) Boron (B) concentration at short time. (**h**) B concentration at long time.

The concentration of Si in SBF proceeded as a function of time after immersion of BaG, and Si release commenced within the first few hours of immersion (Figure 8a). Then, after dissolution of the vitreous network, Si quantity in SBF increased. For all samples, BaG-B_x_/SBF contact time required to reach equilibrium determined after 15 days with a Si concentration in SBF was 65 ppm (Figure 8b). The Si amount released increased tremendously with increased B content in BaG, demonstrating the effect of B on enhancing the BaG-B dissociation process. Variation of Si concentration retained by BaG-B_x_, as a function of B content, was clearly indicated by the shape of obtained curves at short and long test times (Figure 8a,b).

Curves indicate that Si saturation level for BaG-B_20_ is higher than that of BaG-B_0_. Comparing BaG-B_0_ curves with BaG-B_20_ curves demonstrates the effect of B on the intramolecular bond distortion of SiO_4_. This attests to the strong affinity of borosilicate groups (B-O-Si) to SBF solution. Recent research conducted on the interaction of magnesium-doped glass with SBF shows the same Si release behavior from BaG-Mg [79]. Based on molecular dynamics simulations, a bioactive glass ionomer cement (GIC) of different compositions with similar composition and degradability behavior has been generated [80]. Based on the literature [81,82,83], the metabolism of bioactive-glass-forming elements (silicon or boron) comprises clearance by kidneys and elimination through urine.

After immersion of BaG-B_x_ in SBF, Ca concentration in SBF started to rise within few hours and continued to increase over two days (Figure 8c,d). For all BaG-B_x_, concentration of Ca^2+^ ions increased tremendously during the first hours of contact with SBF to reach a value of 150 ppm (Figure 8c). Moreover, with increased B content in BaG, Ca^2+^ release was decreased (as indicated by Ca concentration in SBF) (Figure 8d). This confirms the affinity of Ca ions existing in SBF to the BaG surface [84]. These results agree with the XRD and IR analysis results [85,86,87]. After five days of immersion in SBF, Ca concentration in SBF of the BaG-B_x_ decreased according to both the B content and the immersion time, until equilibrium was reached (Figure 8d). Even after 30 days of immersion, Ca continued to move to the BaG surface, forming a crystallized apatite layer. Weight ratios (e.g., Si/B) can be used to control the release of Ca during in vitro tests [88]. Thus, the Ca amount that moved from SBF to the BaG surface increased with increasing B content of BaG-B_x_. Moreover, Ca concentration in SBF, measured for BaG-B_20_ at 30 days, was much lower than that of boron-free glass (BaG-B_0_, Figure 8d), with a concentration difference:

∆C (ppm) = C_Ca_^B0^ − C_Ca_^B20^ = 20.3 ppm

Calcium ions (Ca^2+^) that were not fully incorporated into the crystallized layer structure that formed on the BaG surface remained in the SBF solution. Consequently, B content of BaG-B is directly related to the rate of Ca^2+^ exchange between BaG and SBF [89].

P ion concentrations in SBF gradually increased due to their continuous release from BaG-B (Figure 8e,f). After 16 h of immersion, P concentrations increased by more than 50 ppm (Figure 8e), indicating a high rate of P release from BaG up to 1 day of immersion, after which P release from BaG-B started to decrease throughout the observation period of 30 days. Starting from two days onward, P was also transferred from SBF to the BaG surface. This is consistent with the precipitation of HAp on the BaG surface. By 30 days, P was completely transferred from SBF to BaG-B (Figure 8f). Higher P transfer to BaG was observed with increased B content in the BaG. Accordingly, it can also be contemplated that B stimulates the transfer of PO_4_^3−^ from SBF to the BaG surface to form an HAp layer (the layer that bonds BaG to bone) [28]. Thus, ICP results showed that two phenomena occur at the BaG-B_x_/SBF solution interface, (1) BaG dissolution in SBF solution; and (2) HAp layer formation confirmed by the migration of Ca and P ions from SBF solution to the BaG surface.

B release from BaG-B to SBF commenced within few hours. BaG-B released between 5 and 11 ppm of B by 16 h of immersion (Figure 8g). Indeed, BaGs with a higher B content dissolved more quickly. By 30 days, 16 ppm B was released from BaG-B_5_, 35 ppm from BaG-B_10_, and 56 ppm from BaG-B_20_ (Figure 8h). The concentration of B in SBF increased with increased B content in BaG-B. This can be explained by increased solubility of BaG-B in physiological fluids with increasing B content [90]. Accordingly, BaG-B with higher B content can suit clinical applications where healing is relatively fast [28], such as occurs in children and upper parts of the body.

## 4. Conclusions

BaG with a high and homogenously distributed B content as well as stable structure can be developed using a modified sol-gel method. The presence of B improves the bioactive glass dissolution in SBF solution by disrupting the silica glass network. The higher the B content of BaG-B, the faster its degradation and B release, as well as ion (Si, Ca, P, B) exchange between the glass and the solution. All studied glasses (5, 10 and 20% B content) were bioactive and exhibited HAp layer formation. The HAp layer of the resulting material becomes denser and thicker as the B content of the material is increased. With these properties, B containing BaG can be useful for application in younger patients and upper parts of the body, where healing is relatively fast and BaG degradation is desired. BaG-B with controlled degradation rate, by controlling its B content, represents an interesting material for future animal and clinical studies that should be carried out.

## Figures and Tables

**Table 1:** Oxide compositions of borosilicate bioactive glasses (BaG-B_x_, wt.%) [47].

BaG	SiO_2_	CaO	Na_2_O	P_2_O_5_	B_2_O_3_
BaG-B_0_	46	24	24	6	0
BaG-B_5_	41	24	24	6	5
BaG-B_10_	36	24	24	6	10
BaG-B_20_	26	24	24	6	20

**Table 2:** Infra-red absorption bands characteristic of hydroxyapatite.

Wave Numbers (cm^−1^)	Assignment	Mode
1121	P-O, antisymmetric elongation of ions PO_4_^3−^	υ_3_
960	P-O, symmetric elongation of ions PO_4_^3−^	υ_1_
603–562	P-O, antisymmetric deformation of ions PO_4_^3−^	υ_4_

**Table 3:** Infrared absorption bands characteristic of silicate groups.

Wave Numbers (cm^−1^)	Assignment	Mode
1235	Si-O	
1045	Si-O-B	
699–794	SiO_4_ on HAp	
458	Si-O-Si	υ_1_
475	SiO_4_ on HAp	υ_2_

**Table 4:** Characteristic infrared absorption bands of carbonate groups.

Wave Numbers (cm^−1^)	Assignment	Mode
1651	CO_3_ on site A et B	
1492	CO_3_ on site B	υ_3_
1410	CO_3_ on site B	υ_3_
873	CO_3_ on site B	υ_2_
